# Supplementary material for: The shift of phosphorus transfers in global fisheries and aquaculture
Source: Nat Commun. 2020 Jan 17;11:355. doi: 10.1038/s41467-019-14242-7 (PMC6969157; doi:10.1038/s41467-019-14242-7)
Supplement: Supplementary file 1 — Supplementary information [file 41467_2019_14242_MOESM1_ESM.docx]

**Supplementary Materials for**

**The shift of phosphorus transfers in global fisheries and aquaculture**

Yuanyuan Huang^1,2*^, Phillipe Ciais^1^, Daniel S. Goll^1,3^, Jordi Sardans^4,5^, Josep Peñuelas^4,5^, Fabio Cresto-Aleina^1^, Haicheng Zhang^1,6^

^1^Laboratoire des Sciences du Climat et de l'Environnement, LSCE/IPSL, CEA-CNRS-UVSQ, Université Paris-Saclay, 91191 Gif-sur-Yvette, France

^2^Commonwealth Scientific and Industrial Research Organisation, Aspendale, 3195, Victoria, Australia

^3^Department of Geography, University of Augsburg, Germany

^4^CSIC, Global Ecology Unit CREAF-CSIC-UAB, 08913 Bellaterra, Catalonia. Spain

^5^CREAF, 08913 Cerdanyola del Vallès, Catalonia, Spain

^6^Department Geoscience, Environment and Society, Université Libre de Bruxelles, 1050, Bruxelles, Belgium

**Corresponding author**: Yuanyuan Huang

Email: yuanyuanhuang2011@gmail.com

**Supplementary Methods**

**Boundary conditions.** The fishery industry involves numerous activities that are directly or indirectly linked to P dynamics. This study focuses on the major flows between aquatic ecosystems and land that are directly perturbed by fishery activities. We list the boundaries for historical P budget quantification as follows:

Boundary 1, fishery P flow between the aquatic environment and land. We do not consider P transferred between wild fishery and aquaculture; for example, through escaped fish from fish farms or lost feed. Nor do we consider transfer between different sectors of land systems — these are treated as exchanges within each system. Similarly, P transfer associated with water used to raise fish is P transfer within aquatic ecosystems.

Boundary 2, fishery P flow that results from deliberate activities involving raising and harvesting fish. P flow associated with activities such as water discharged from wastewater treatment plants, leached from agriculture soils, or contributed from other point or non-point sources that indirectly benefit wild fish growth, is not treated as a deliberate activity that aims to raise or harvest fish. On the other hand, point or non-point P sources that are purposely directed into aquaculture, such as the sewage-fed aquaculture[^1^](#_ENREF_1)^,^[^2^](#_ENREF_2), are accounted for in our P budgeting through fish feeds and fertilizers.

Boundary 3, P flow where fishery plays the major role from the management perspective. As shown in Supplementary Fig. 1, P transfers among different sectors are complex as P recycles through vegetation, livestock, human society, aquaculture, wild fishery and waste disposal systems. Because of the limited reliable data available, this study does not aim to conduct life-cycle assessments that track every detail of these processes. Harvested P that ends up in the aquatic environment through feeding or fertilizing aquaculture is a direct transfer that is mediated by fishery management. We do not consider harvested P that re-enters natural aquatic ecosystems indirectly, for example, through human waste sewage systems or leaching from fertilized cropland. Waste management plays a bigger role in the former case and agriculture is responsible for the latter.

Miscellaneous. The discharge of P from cruise ships is assumed to be trivial and not tracked in this study. We do not subtract the biomass of larval and juvenile fish, assuming the biomass of larval and juvenile fish is relatively small compared to the total fish weight and the gap in biomass is well covered by uncertainties in fish production database. Despite these omissions, we believe our current approach is a reasonable first order approximation, we acknowledge P-input estimated from this approach is different from the final P loading that reaches the ocean. In pond aquaculture, the impact of P is limited within the pond if water is not discharged. In some ponds, water or sediments are reused to fertilize crops which is likely to increase P-use efficiency of integrated crop-livestock-aquaculture systems. We do not explicitly account for this flux, as this practice is spontaneously carried out by farmers with small ponds.


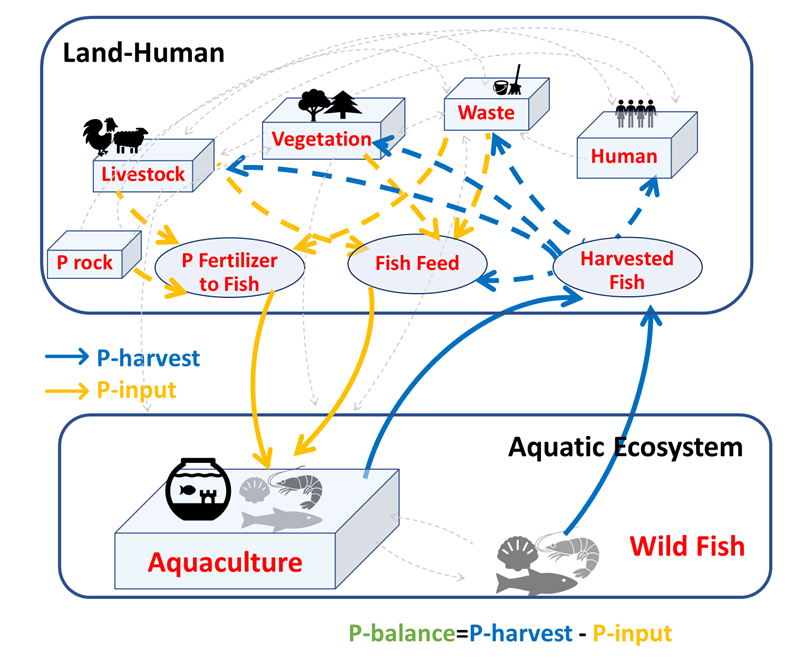


**Supplementary Fig. 1. Fishery P flows.** The figure provides a more detailed view of P transfers involving fishery, as a supplement to Fig. 1 in the main text. Fishery is a collection of diverse activities involving raising and harvesting fish. Harvested fish transports P from aquatic ecosystems to land (solid blue arrows). The fate of harvested fish P varies―some P is returned to aquatic ecosystems through feeding aquaculture with fishmeal and oil; some P is consumed by humans and enters the sewage system; some P ends up in landfill, while some P is used to feed livestock or fertilize crops (dashed blue arrows). Fish farming requires P supply primarily through fish feed and P fertilizer (solid orange arrows). Fish feed formulation ranges from manufactured compound feeds and trash fish, to products or residues from crops, livestock and human food wastes. P fertilizer includes manure and mineral fertilizers. Fishery P transfers involve complex interactions among terrestrial vegetation, livestock, human society and waste managements (dashed grey arrows). This study focuses on the major perturbation of P flows between aquatic ecosystems and land caused by fishery. We adopt a land or human-centric viewpoint. We call external P that goes directly into the aquatic environment from fishery, P-input, and P that moves out of the aquatic ecosystems through harvested fish, P-harvest. We use P-net that is the difference between P-harvest and P-input, to quantify the net P budget between aquatic ecosystems and land. Details of the boundary conditions are provided in the text of the supplementary section.

**Supplementary Discussion**

**Data pattern**. For fish biomass production, Based on FishStatJ 3.04.6, annual wild capture of fish peaked at 94 Tg and has stayed relative static since the late 1990s, while aquaculture fish production increased rapidly, from 0.6 Tg in 1950 to 80 Tg in 2016 (Supplementary Fig. 2). Finfish dominate the wild capture production and are also responsible for more than 50% of the aquaculture fish production. The relative contribution of finfish to aquaculture production is generally increasing with time, which is the opposite to mulluscs. Crustaceans constitute a non-trivial portion in aquaculture production, which is increasing with time (Supplementary Fig. 2).


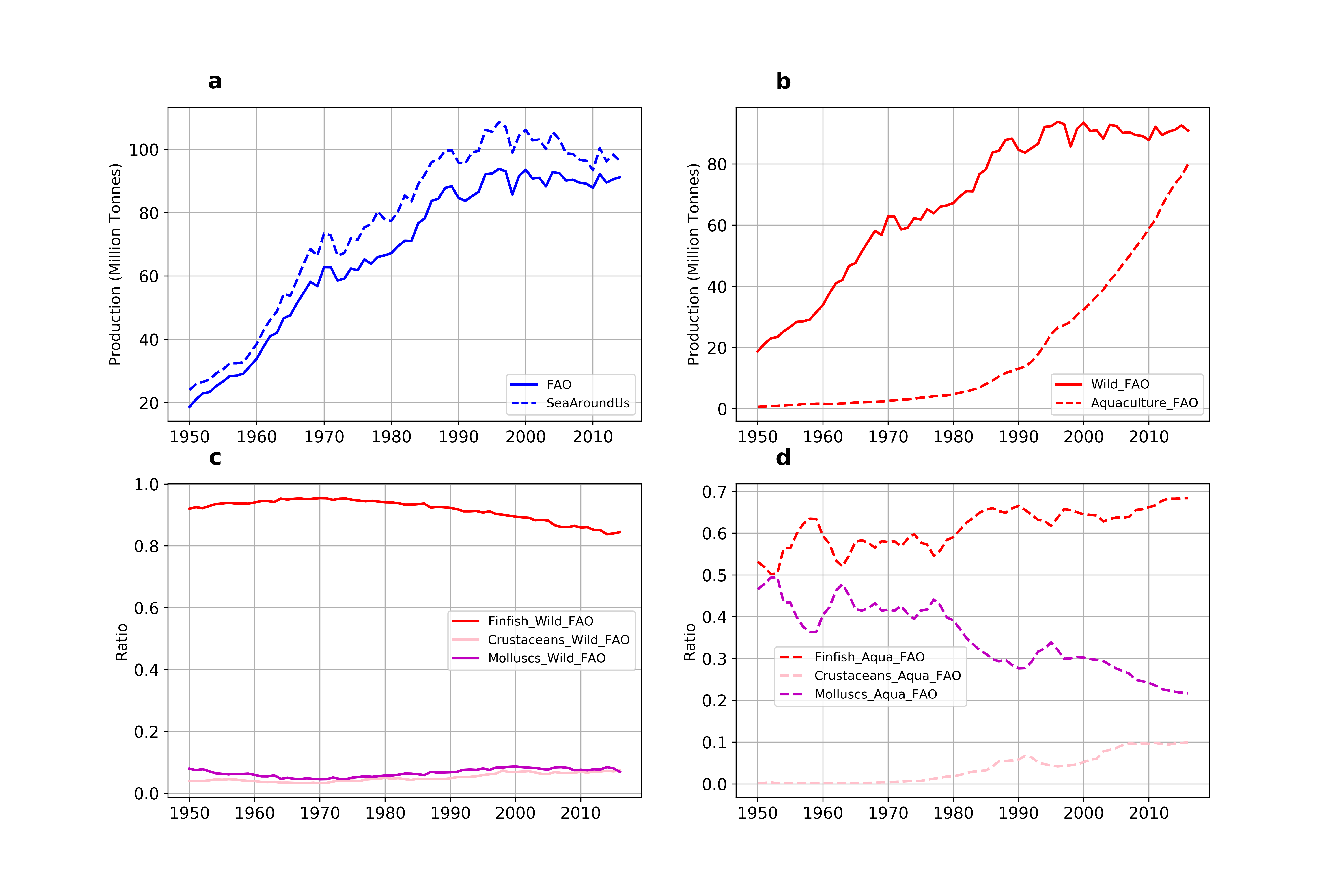


**Supplementary Fig. 2. Global fish biomass production.** Wild capture fish production from FAO and SeaAroundUs (**a**); wild capture and aquaculture fish biomass from FAO database (**b**); relative contributions from different fish groups (finfish, crustacean and mollusc) for wild capture fishery (**c**) and aquaculture (**d**)

For Fish P concentration, Finfish generally have a higher median P concentration (0.75% under wild and 0.53% under farmed conditions) than crustaceans (0.32% for wild and 0.32% for raised) and mollusks (0.13% for wild and 0.15% for raised, including both soft tissue and shell) (Supplementary Fig. 3). The central P concentration is likely to be biased towards species that have higher scientific or economic values and appear more frequently in studies. Alternatively, the median of species-average (one value for each species) P concentration is 0.75% for wild finfishes, 0.66% for farmed finfish, 0.31% for wild crustaceans, 0.24% for raised crustaceans, 0.24% for wild mollusks and 0.14% for raised mollusks (Supplementary Fig. 4).


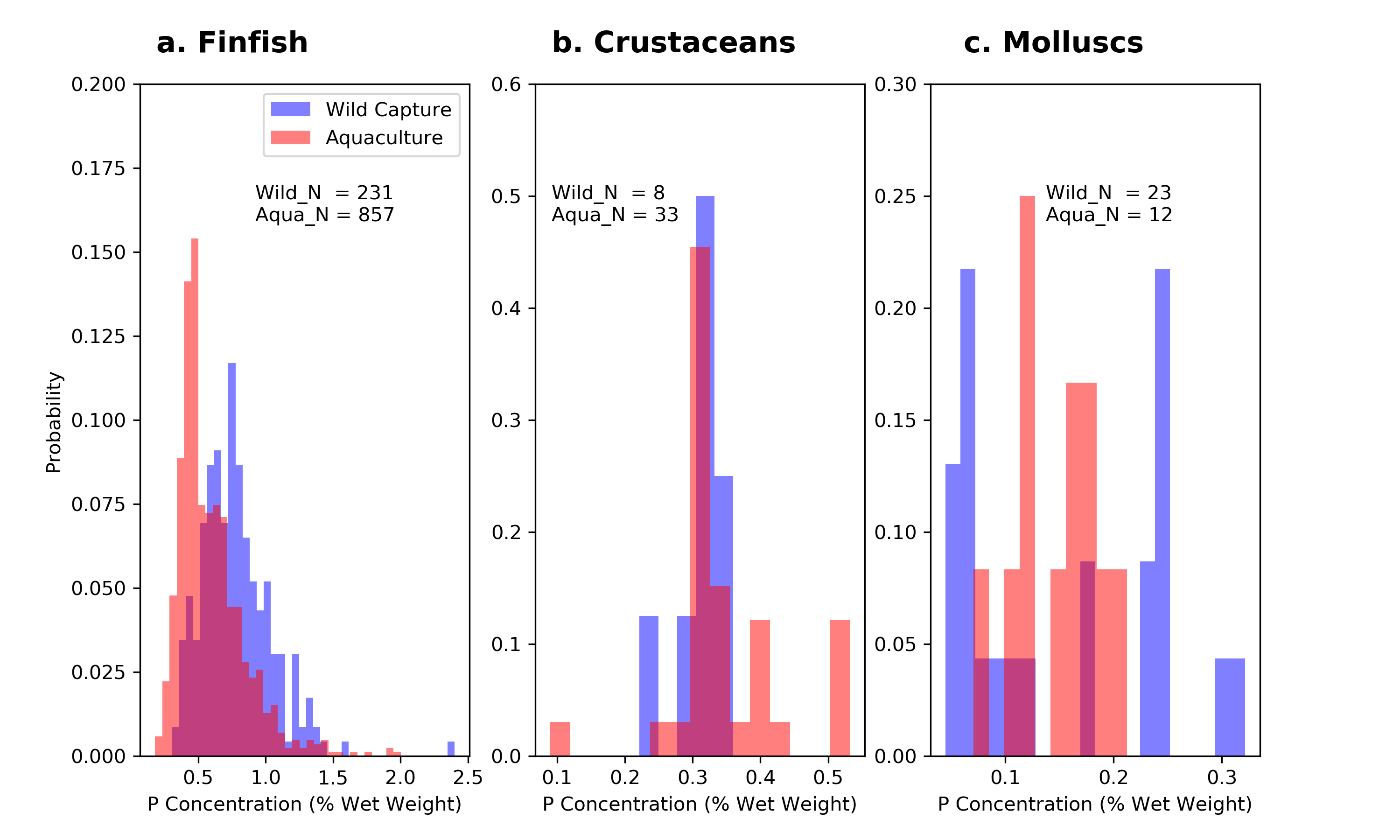


**Supplementary Fig. 3. Distribution of fish whole-body P concentration across the entire database.** Numbers correspond to individual studies. The entire database covers 224 fish species.


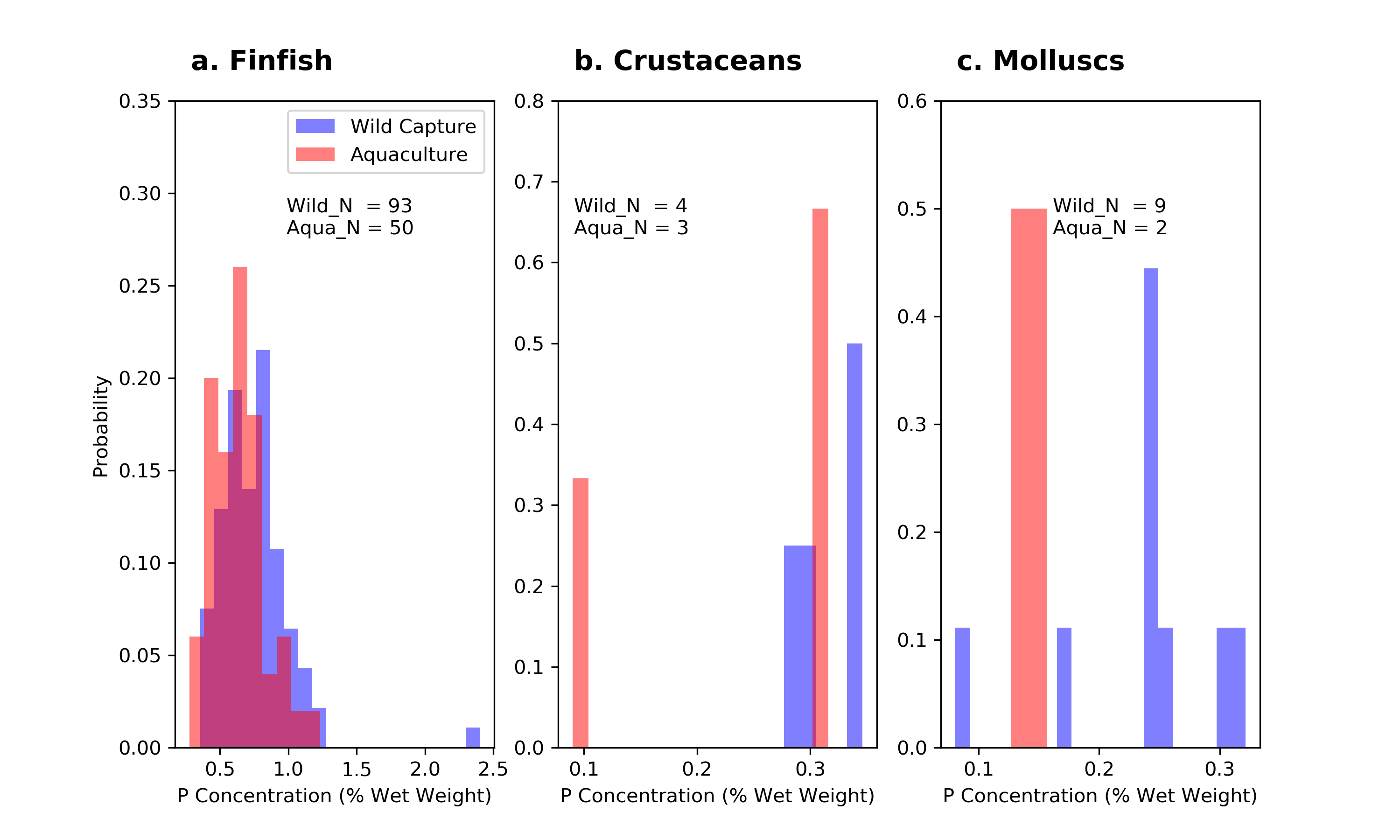


**Supplementary Fig. 4. Distribution of species level fish whole-body P concentration.** Numbers correspond to the total number of species.

For Culture-system P use efficiency, culture-system level PUE ranges from 1% to 167% with a median of 15% across the PUE database (Supplementary Fig. 5). A PUE larger than 100% indicates the utilization of P pre-existing in the surrounding environment in addition to external P additions. This process may occur with non-fed species, omnivores or polyculture systems that benefit from P-rich waters. An extremely low PUE corresponds to low efficient resource management, for example, with extensive culture practice. Finfish farming has a significantly (*P < 0.01*) higher PUE than the cultivation of crustacean species (e.g., crabs, lobsters, crayfish, shrimp). The median PUE of freshwater finfish farming is 18% and marine finfish farming is 27%, while the PUE is 11% for both fresh and marine crustacean cultivation (Supplementary Fig. 5). The median of finfish PUE is 19% in China, higher than 11% in India, 11% in Vietnam and 14% in Thailand. Crustacean species farming systems have a median PUE of 12% in China, 11% in India, 10% in Bangladesh and 11% in Thailand.


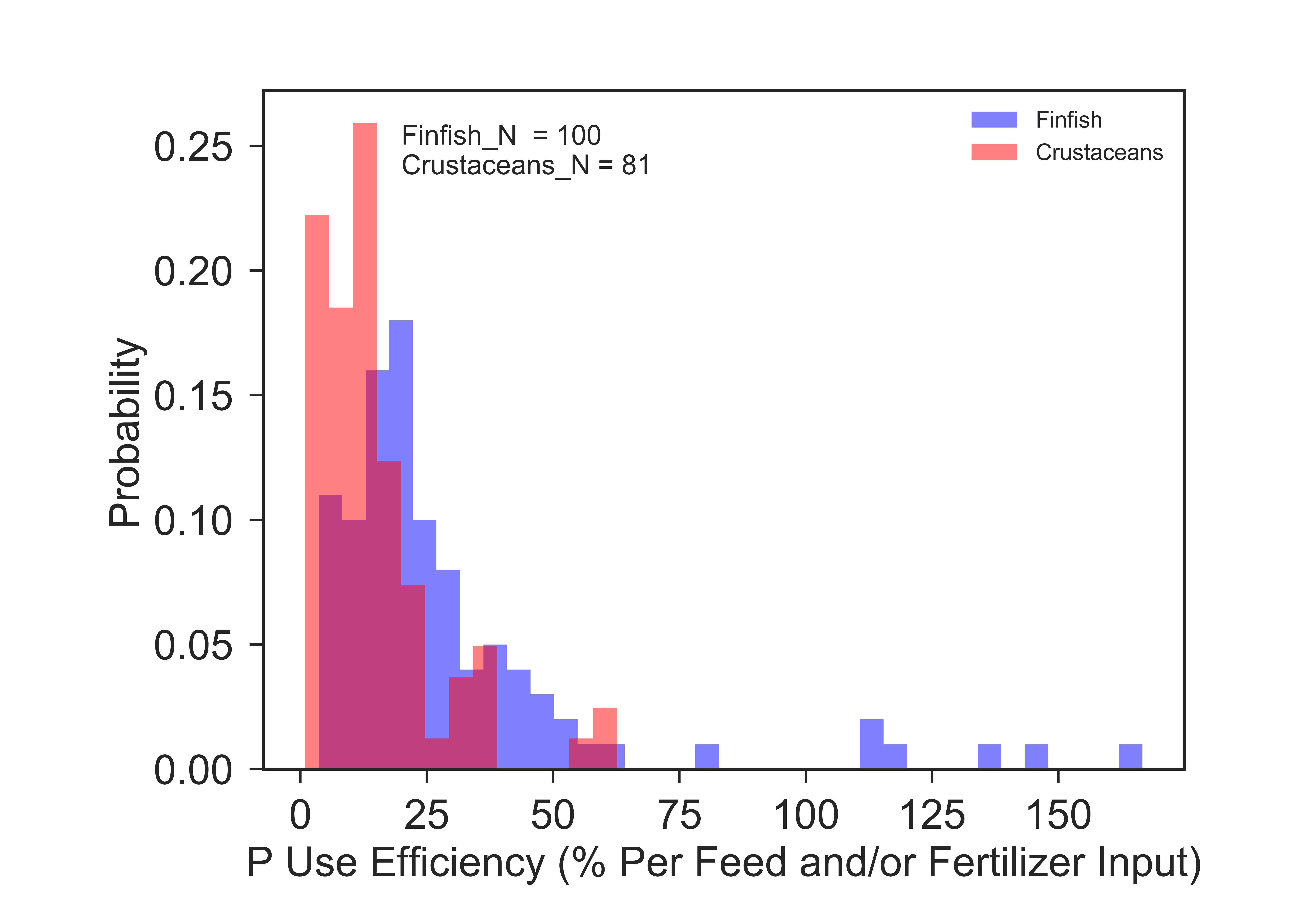
 **Supplementary Fig. 5. Distribution of culture-system level P use efficiency.** Numbers correspond to individual studies.

For P retention from feeding experiments, P-retention efficiency (PRE) is the fraction of P that is recovered in fish biomass per unit of feed P intake from feeding experiments conducted in well-controlled environments (e.g., tanks) with different treatments. We compiled a data set of fish P retention efficiency from the literature (Supplementary Fig. 6). PRE ranges from 1% for Japanese flounder to 103% for one rainbow trout feeding experiment. The mean PRE is 40% and the median PRE is 37% across the entire PRE records. Carp takes a share of ~40% of total aquaculture production by weight and their median PRE reaches 44%. Median PRE of other important cultivated species is: cod, 60%; turbot, 57%; catfish, 47%; porgy, 44%; trout, 44%; salmon, 38%; flounder, 37%; croaker, 35%; seabream, 34%; seabass, 34%; tuna, 31%; tilapia, 28%; sturgeon, 23%; shrimp, 21%; sole, 18% and milkfish, 11%.


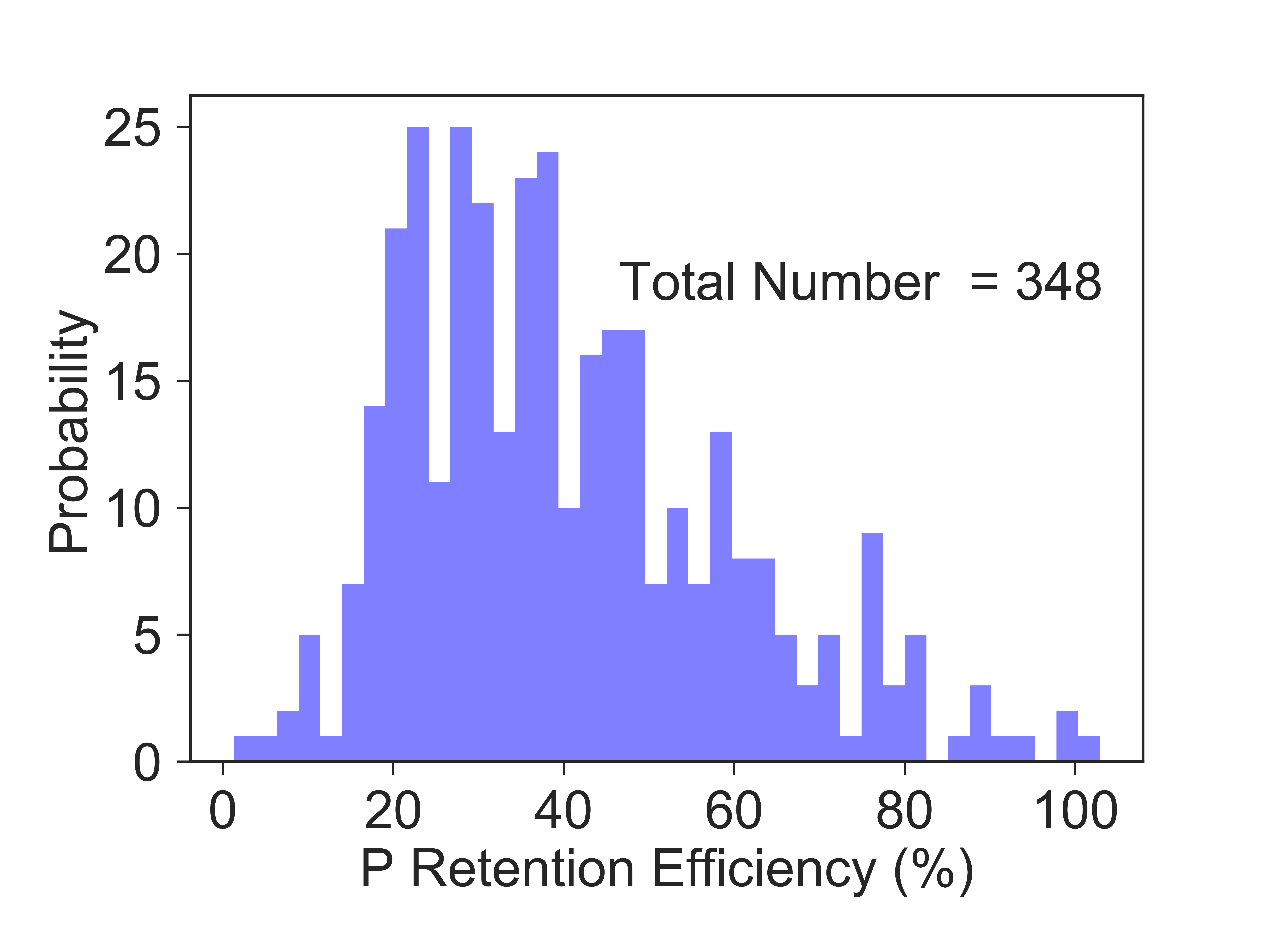


**Supplementary Fig. 6. Distribution of P-retention efficiency from feeding experiments.**

**Harvested P to be used by land activities in 2010.** We calculate the amount of harvested P that can be further recaptured by land-based human activities through subtracting from total harvested P by the amount of P that ends up as fishmeal and fish oil. Here, we document the calculation for year 2010, because for this year we found data on global fishmeal and fish oil production as well as the portion used as aquaculture feed. In 2010, total P-harvest is 0.98 Tg P. With 18.5 Tg (live weight equivalent) of captured forage fish being used to produce fishmeal and fish oil in 2010[^3^](#_ENREF_3), and with a global average P concentration of 0.76% from our data, we estimated that 0.14 Tg P ends up in fishmeal and fish oil. Harvested P that could be further recaptured by land activities, excluding fishmeal and fish oil, is therefore 0.84 Tg P (0.98 minus 0.14). We separated P in fishmeal and fish oil into two fractions: one that goes as feed to aquaculture and the other that serves as food/supplementary for human and land animals. In 2010, aquaculture consumed 73% of fishmeal with the rest being fed to poultry and swine and other sectors3. For fish oil, we only found statistics for 2012 when 74% of fish oil was reported to be used by aquaculture with the rest going to direct human consumption or being used in other sectors4. We therefore assumed that 73% of fish oil was applied to aquaculture in 2010 to simplify the calculation. The total harvested P that directly goes back into aquaculture through fishmeal and fish oil is 0.10 Tg P (73% x 0.14), leaving 0.4 Tg P being used by human and land animals.

**Relative contributions to P budget from different fish groups.** Across different fish groups, finfish dominate P-harvest from both wild capture (94–98%) and aquaculture (83–90%) (Supplementary Fig. 7), and P-input into both fresh and marine water aquaculture (Supplementary Fig. 8). For wild capture, the proportion of finfish has been decreasing slightly with time, while both crustaceans and molluscs have been increasing with time. For aquaculture, there is no clear temporal trend in finfish, but the proportion of crustaceans has been increasing with time, compensating the reductions in the proportion of molluscs. The majority of aquaculture P-input comes from freshwater, with the contribution ranging from 84–94% and slightly decreasing with time. Within the freshwater aquaculture, P-input comes exclusively from finfish (94.58–99.99%), and the share of crustaceans slightly increases with time, to reach 5.31% in 2010. Within the marine aquaculture, most of P-input (> 90%) ends up in finfish aquaculture during 1950–1970; however, after 1990, around 50% of marine aquaculture P-input goes into raising crustacean species.


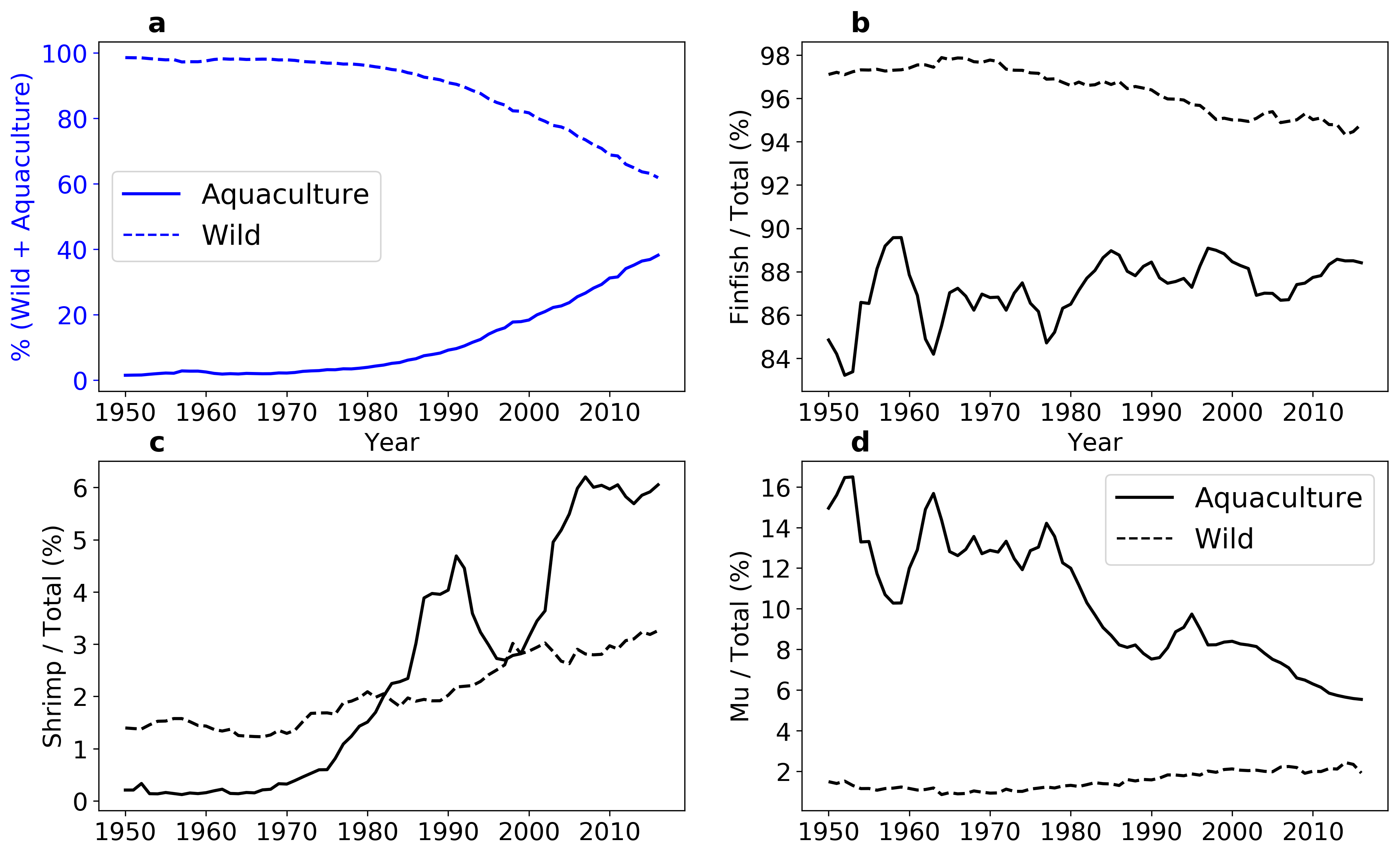


**Supplementary Fig. 7. Relative contributions to total P-harvest.** **a,** the percentage contribution of wild capture (dashed line) and aquaculture (solid line) to the total P-harvest and **b**, the percentage contribution of finfish to total fishery P-harvest. **c**, the percentage contribution of crustacean (shrimp) to total fishery P-harvest. **d**, the percentage contribution of molluscs to total fishery P-harvest. Solid lines are from aquaculture and dashed lines from wild capture.


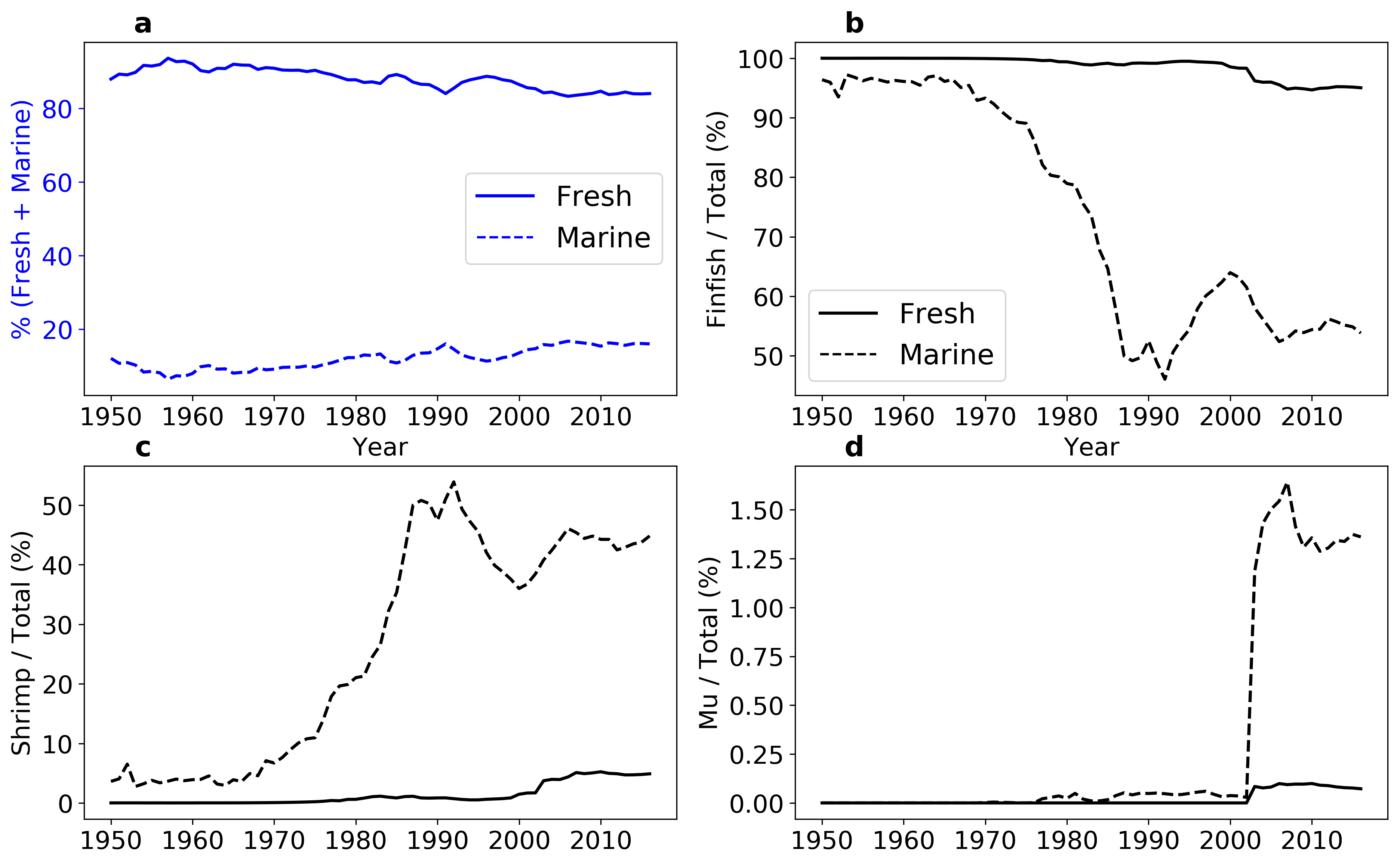


**Supplementary Fig. 8. Relative contributions to total fishery P-input.** **a,** the percentage contribution of fresh (solid line) versus marine (dashed) aquaculture to the total global fishery P-input and **b**, the percentage contribution of finfish to total fishery P-input. **c**, the percentage contribution of crustacean (shrimp) to total fishery P-input. **d**, the percentage contribution of molluscs to total fishery P-input. Solid lines are from fresh water aquaculture and dashed lines from marine aquaculture.

**Uncertainty and sensitivity.** P-harvest is not sensitive to the way we draw random samples from the whole-body P concentration database. When the calculation shifts to rely more on order and group level information (10 compared to 1), changes in both 95% and 5% percentile of 1000 Monte Carlo calculations are less than 1% (Supplementary Fig. 9c). P-input is more sensitive to P concentration sampling. The 95% percentile is on average lowered by 6% and the 5% percentile is 4% smaller when the random sampling relies more on order level information. Species level samples capture the physiology that drives variations in fish P concentration, but limited sampling size may fail to capture variations of P concentration under different environments. Nevertheless, the sampling strategy has very limited impact on P-harvest estimation for both wild capture (<1%) and aquaculture ( < 1%). The bigger impact on P-input is related to the high sensitivity of P-input to PUE, the skewed distribution of PUE and the complexity raised through uncertainty.

When the uncertainty level of fish production is ±50%, the 95% percentile of P-harvest is 4% higher compared to no uncertainty in fish production, and the 5% percentile is 3% lower (Supplementary Fig. 10). P-input is less sensitive to a 50% uncertainty in fish production, with 1% differences in both the 95% and 5% percentiles. Based on the IPCC criteria, 50% percentile uncertainty represents very low confidence[^5^](#_ENREF_5). Practically, a ±100% percentile uncertainty is not commonly considered. For testing purposes, a ±100% percentile uncertainty in fish production expands the upper percentile level by 11% and the lower by 10% in P-harvest, while there is only a 2% difference in both the upper percentile and lower percentile from P-harvest (Supplementary Fig. 11). The low confidence in fish production does not strongly affect the uncertainty of P budget estimation due to the relatively large variation in whole-body P concentration and PUE for estimating P-harvest.

P-harvest is sensitive to PUE. P-net in finfish aquaculture was -0.38 Tg in 2010, which is larger than the -0.1 Tg estimated from Ref[^6^](#_ENREF_6). Ref[^6^](#_ENREF_6) modelled PUE through feed conversion ratio (FCR, feed biomass: fish biomass) and P fraction in feed for major finfish species. PUE from Ref [^6^](#_ENREF_6) (33% for rainbow trout, 19% for gilthead seabream, 25% for eel, 34% for catfish, 22% for catla, 36% for tilapia) is generally higher than the median culture-system level PUE compiled from this study (Supplementary Fig. 5). PUE of super intensive, intensive, specialized semi-intensive, specialized semi-extensive, integrated semi-intensive, integrated semi-extensive culture system is 9%, 15% 12%, 30%, 17%, 3% respectively derived from a survey of 2493 farms across nine Asian countries[^7^](#_ENREF_7), pointing to a large scope for improving P resource management, especially in developing Asian countries.


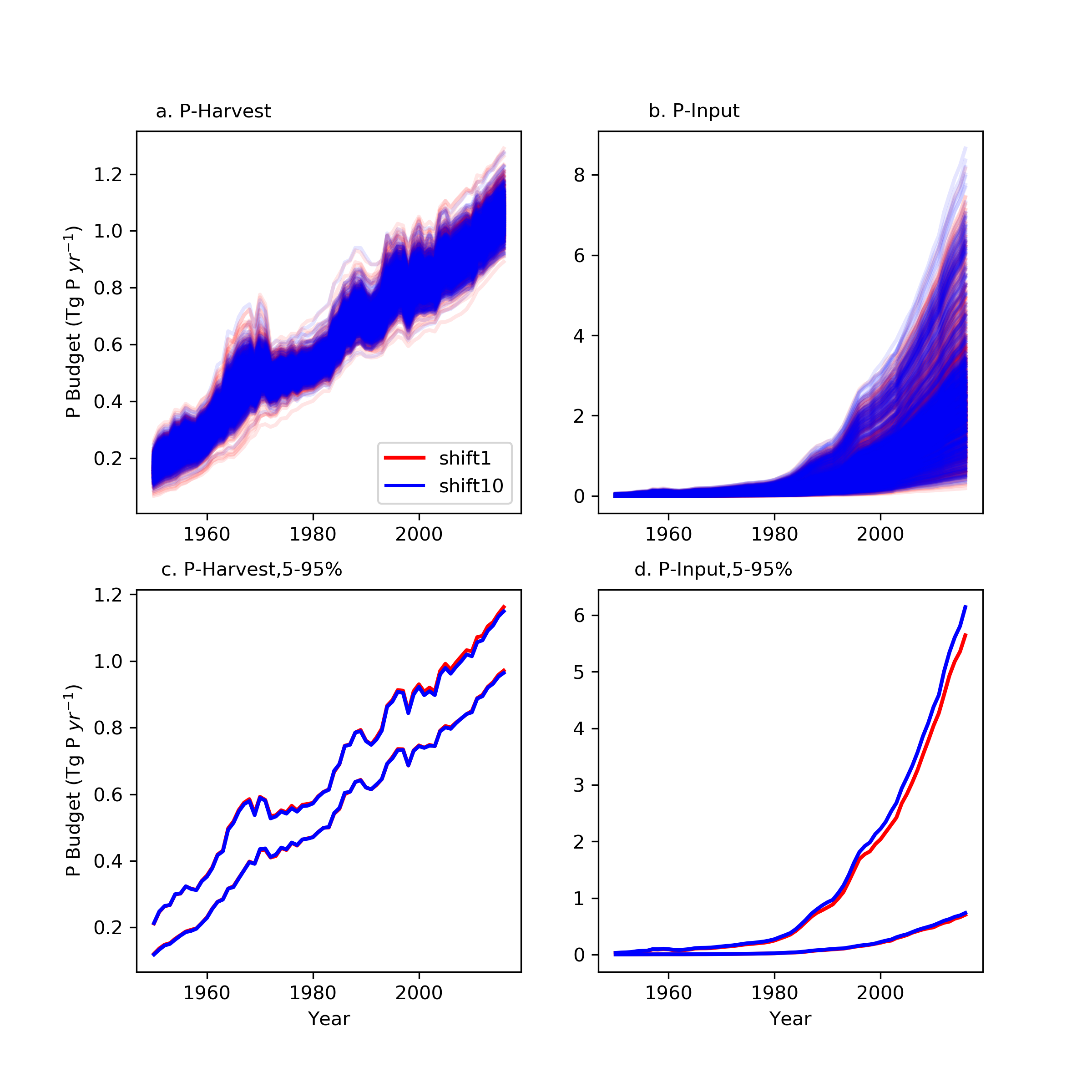


**Supplementary Fig. 9. Uncertainties of P budget estimations caused by sample size criterion.** **a** and **b** are results from 1000 Monte Carlo calculations for P-harvest and P-input respectively, while **c** and **d** are their 5%-95% quantile ranges. Blue indicates the calculation with 1 as the criterion on when to shift to rely on order (or group) level information. Red lines represent calculations that rely on order (or group) level information when the corresponding entry in the compiled P concentration database is no more than 10.


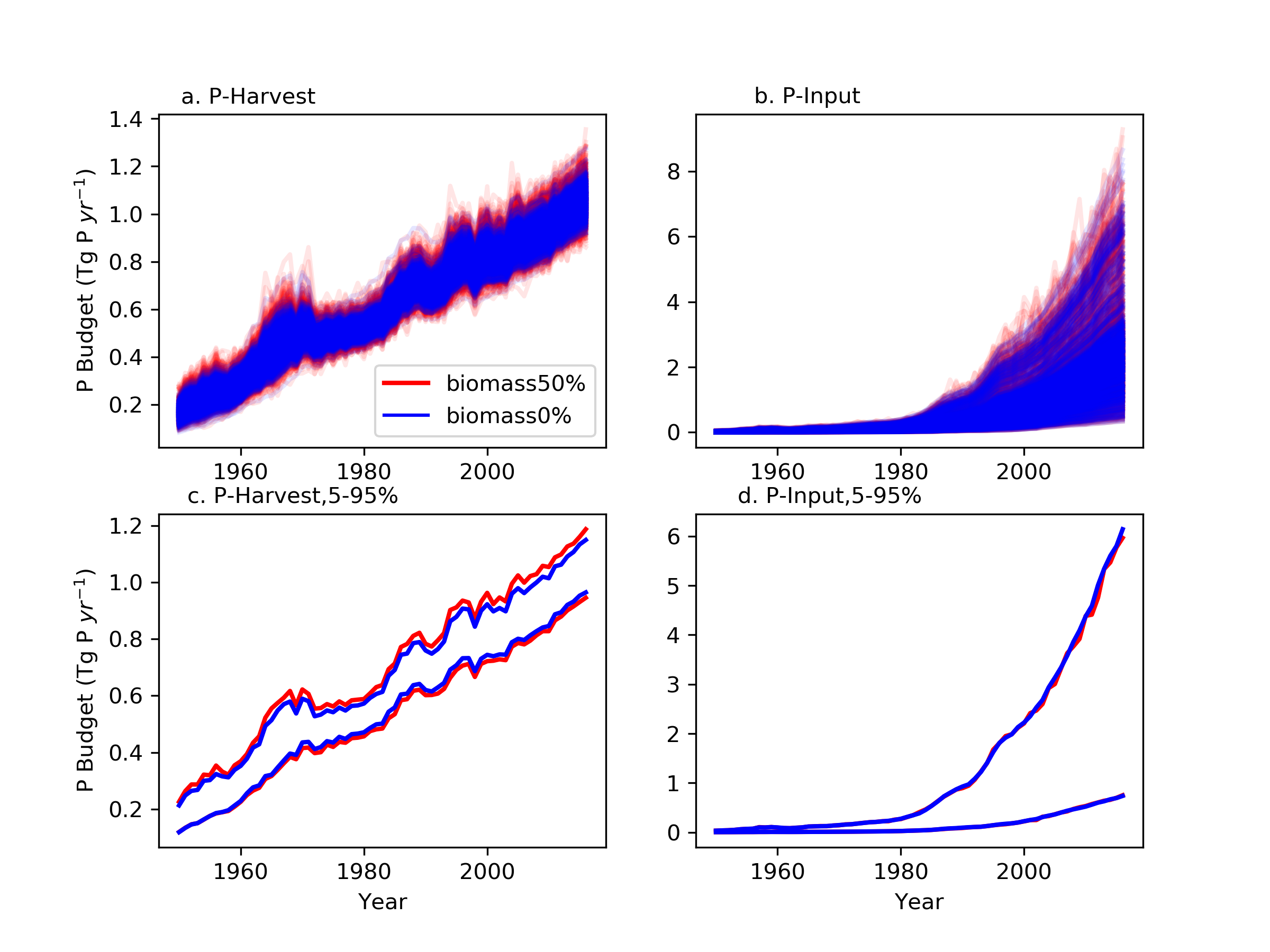


**Supplementary Fig. 10. Uncertainties of P budget estimates caused by fish biomass uncertainty.** **a** and **b** are results from 1000 Monte Carlo calculations for P-harvest and P-input respectively, while **c** and **d** are their 5%-95% quantile ranges. Blue lines indicate calculations with 0% uncertainty in fish biomass and red corresponds to ± 50% percentile uncertainty.


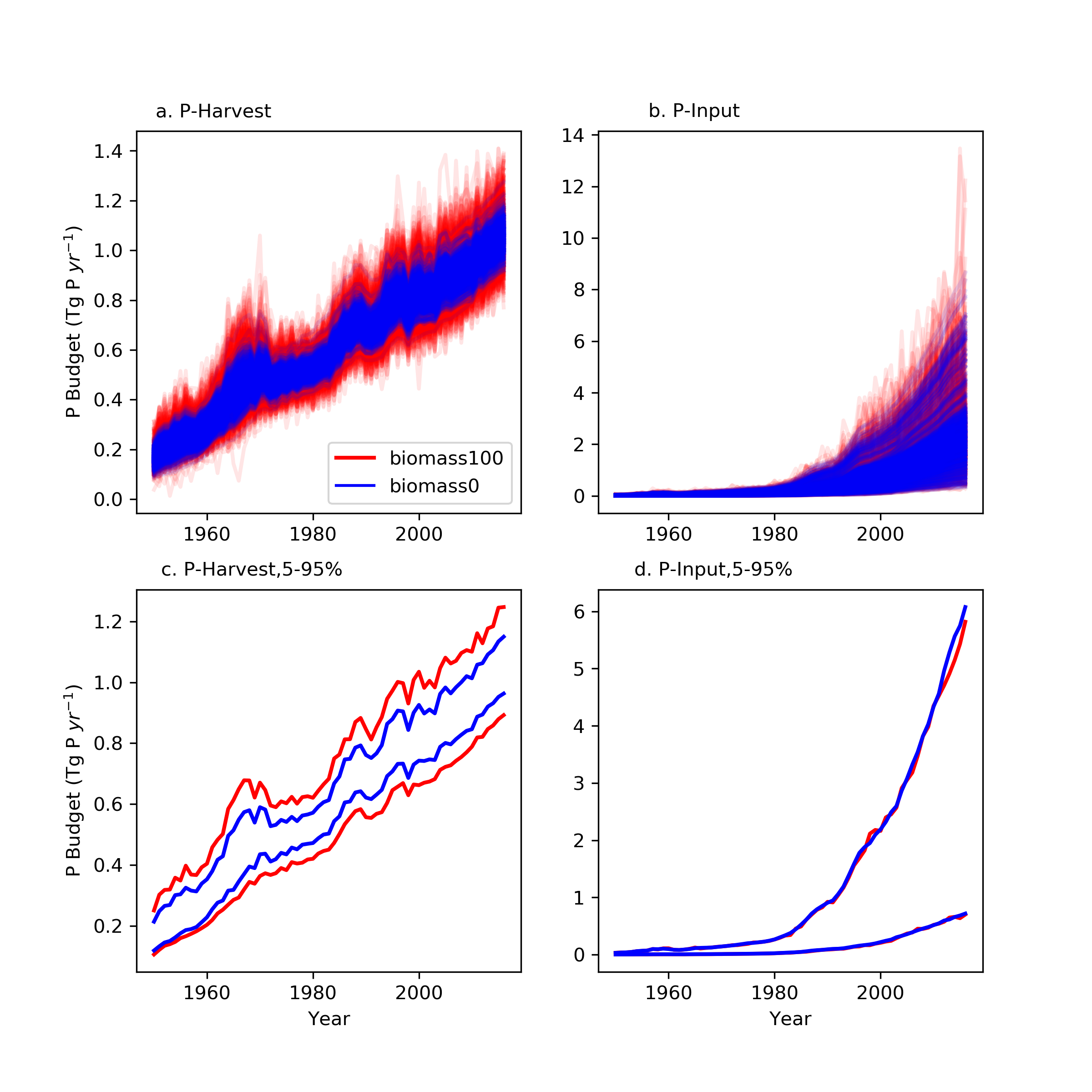


**Supplementary Fig. 11. Uncertainties of P budget estimations caused by fish biomass uncertainty.** **a** and **b** are results from 1000 Monte Carlo calculations for P-harvest and P-input respectively, while **c** and **d** are their 5%–95% quantile ranges. Blue lines indicate calculations with 0% uncertainty in fish production and red corresponds to ±100% percentile uncertainty.

**Country level P budget.** In the 1970s, most countries contributed to the positive P-net (Supplementary Fig. 12). However, in the most recent decade, the negative P-net from developing Asian countries dominate the global P-net, this occurs despite most developed countries being characterized by a positive P-net (Supplementary Fig. 13). Despite an overall reduction in global P-net in the most recent decade, countries such as Russia, United States, Morocco, Iceland, Namibia, Mexico, New Zealand, Argentina, Mauritania and Sri Lanka, are all characterized by increases in P-net (Fig. 3). However, the total increase is only 9% of the reductions — resulting in a global reduction in P-net. Among countries that show a reduction in P-net, the top 10 countries, i.e., China, India, Vietnam, Japan, Indonesia, Bangladesh, Thailand, Egypt, Brazil, Norway and Denmark, account for 91% of the total reduction.





**Supplementary Fig. 12. Average annual country level P budget in the 1980s (1980-1989).** Panel **a** is P-harvest and **b** is P-input. Panels **c** and **d** represent the same P-net (P-harvest - P-input) with different color schemes. Panel **c** uses the categorized colour scheme and panel **d** is on a log scale. Note the units are 0.01 Tg P yr^-1^. Country borders are adapted from TM World Borders Dataset 0.3





**Supplementary Fig. 13. Average annual country level P budget in the most recent 10 years (2007-2016).** The colour scheme is the same as in Supplementary Fig. 12. Country borders are adapted from TM World Borders Dataset 0.3.


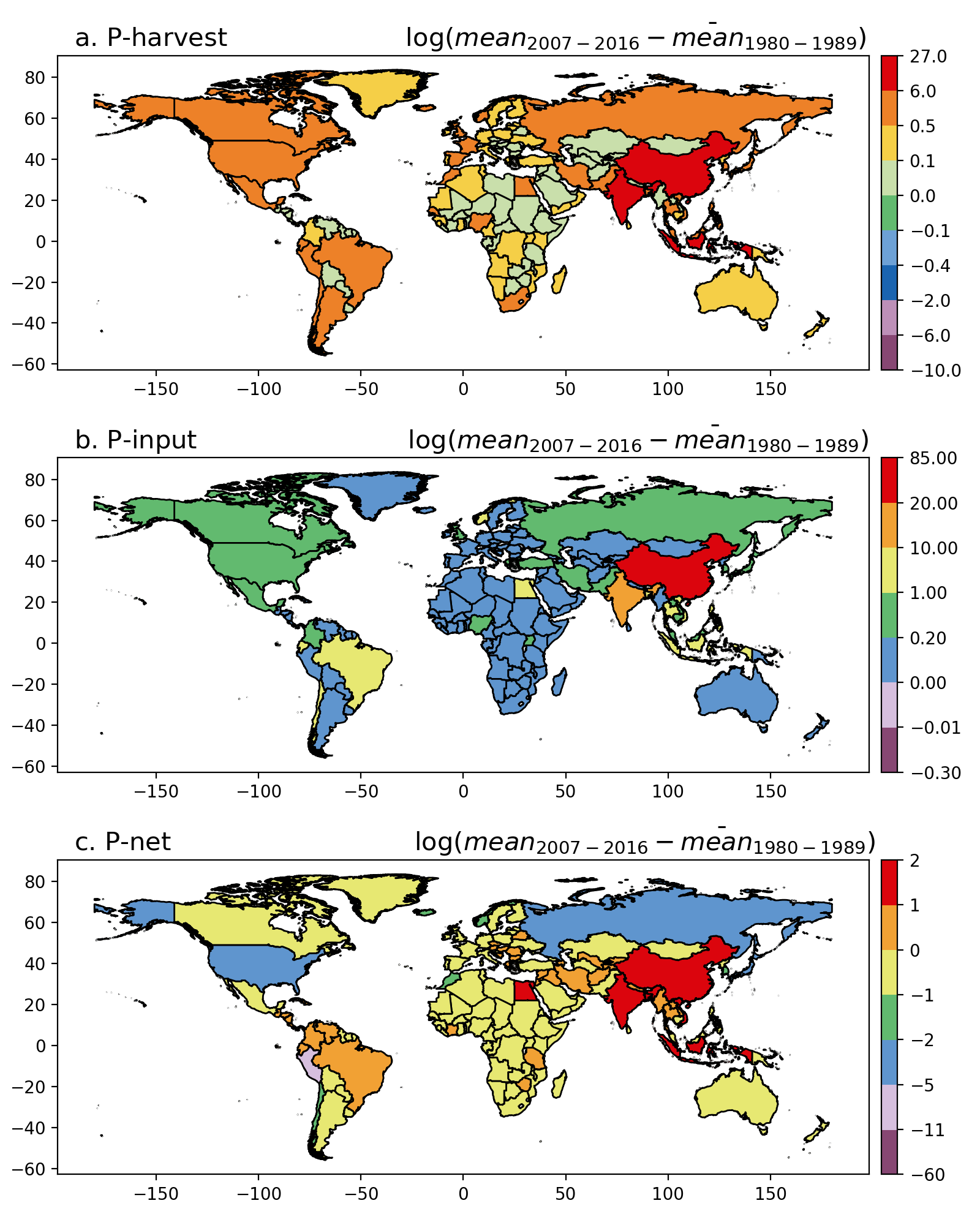


**Supplementary Fig. 14.** **Changes in the global fishery P budget.** The same as the Fig. 4 except for the differences in the colour scales and units here are 0.01 Tg P yr^-1^. Country borders are adapted from TM World Borders Dataset 0.3.

**Comparing aquatic vs. terrestrial P budget (per unit protein supply).** We compare here the contribution of capture fisheries and aquaculture vs. terrestrial food production sectors (crop and livestock) to aquatic P loading per unit protein supply. Global total food protein supply was estimated through multiply the per capita protein supply quantity by the global population. Per capita protein supply quantity was obtained from FAOSTAT food balance sheet (<http://www.fao.org/faostat/en/#data/FBS>, accessed on 17^th^ September, 2019) which tracks protein supply from 1961 to 2013. The world population data was from the World Bank (https://data.worldbank.org/indicator/sp.pop.totl, accessed on 17^th^ September, 2019). Total P loading from crop and livestock into aquatic environment varies among studies. Total P loading into aquatic ecosystems through crop-livestock varies among studies and studying year. [Bouwman, et al. ^8^](#_ENREF_8) reported a value of 4 Tg P yr^-1^ in 2000 driven by fertilizer and manure input into terrestrial food productions. The synthetic study from [Peñuelas, et al. ^9^](#_ENREF_9) reported a value of 5 Tg P yr^-1^ (2005-2011) from fertilizer leaching and erosion, and 13.5-25 Tg P yr^-1^ if taking into account land use change driven largely by agriculture expansion. From [Chen and Graedel ^10^](#_ENREF_10), 1.7 Tg P yr^-1^ entered water bodies through leaching of agriculture soil P, 9.0 Tg P yr^-1^ from agriculture soil through runoff, 0.7 TgP yr^-1^ from crop residue, 1.4 TgP yr^-1^ from animal waste, that is, 12.9 Tg P was transferred into water bodies from the crop-livestock system in 2013. [Lun, et al. ^11^](#_ENREF_11) documented that 9.7 TgP yr^-1^ (cropland runoff: 3.7 TgP yr^-1^; Pasture runoff: 1.7 TgP yr^-1^; and manure waste: 4.3 TgP yr^-1^) ended up in non-agricultural land or body of water during 2002-2010. Based on P budgets listed above and fishery P budget from this study, Tab. S1 compares P budget per unit food protein supply driven by fish vs. crop and livestock productions.

**Supplementary Tables**

Supplementary Table 1. Global P and protein budgets from different food production sectors. The positive sign for P in the table corresponds to load into the aquatic ecosystem and the negative sign means an extraction of P from aquatic ecosystems.

|  | **Capture Fisheries and aquaculture** | | | **Aquaculture** | | | **Terrestrial food production**  **(crop + livestock )** | | |
| --- | --- | --- | --- | --- | --- | --- | --- | --- | --- |
| Year | P  (Tg yr^-1^) | Protein  (Tg yr^-1^) | P per Protein (g g^-1^) | P  (Tg yr^-1^) | Protein  (Tg yr^-1^) | P per Protein  (g g^-1^) | P  (Tg yr^-1^) | Protein  (Tg yr^-1^) | P per Protein  (g g^-1^) |
| 2000 | -0.19 | 9.87 | -0.019 | 0.56 | 1.81 | 0.310 | 4^ref8^ | 158 | 0.025 |
| 2005-2011 | 0.31 | 12.4 | 0.025 | 1.01 | 3.5 | 0.290 | 5^ref9^  13.5-25^ref9^ | 181 | 0.028  0.074-0.138 |
| 2002-2010 | 0.17 | 11.67 | 0.015 | 0.88 | 3.01 | 0.293 | 9.7^ref11^ | 174 | 0.056 |
| 2013 | 0.71 | 13.67 | 0.052 | 1.40 | 4.80 | 0.292 | 12.9 ^ref10^ | 199 | 0.065 |

Supplementary Table 2. Geo-region designation from FishStatJ version 3.04.6 (FAO, 2018).

| **GeoRegion** | **Name_En** | **GeoRegion** | **Name_En** | **GeoRegion** | **Name_En** | **GeoRegion** | **Name_En** |
| --- | --- | --- | --- | --- | --- | --- | --- |
| Australia and New Zealand | Australia | Eastern Africa | Zimbabwe | Northern Europe | Ireland | Southern Europe | Croatia |
| Australia and New Zealand | Christmas Island | Eastern Africa | South Sudan | Northern Europe | Latvia | Southern Europe | Gibraltar |
| Australia and New Zealand | Cocos (Keeling) Islands | Eastern Africa | Uganda | Northern Europe | Lithuania | Southern Europe | Greece |
| Australia and New Zealand | Heard and McDonald Is. | Eastern Africa | Tanzania, United Rep. of | Northern Europe | Norway | Southern Europe | Holy See |
| Australia and New Zealand | New Zealand | Eastern Africa | Zanzibar | Northern Europe | Sark | Southern Europe | Italy |
| Australia and New Zealand | Norfolk Island | Eastern Africa | Zambia | Northern Europe | Svalbard and Jan Mayen | Southern Europe | Malta |
| Caribbean | Antigua and Barbuda | Eastern Asia | China | Northern Europe | Sweden | Southern Europe | Montenegro |
| Caribbean | Bahamas | Eastern Asia | Taiwan Province of China | Northern Europe | United Kingdom | Southern Europe | Portugal |
| Caribbean | Barbados | Eastern Asia | China, Hong Kong SAR | Northern Europe | Channel Islands | Southern Europe | San Marino |
| Caribbean | British Virgin Islands | Eastern Asia | Japan | Northern Europe | Guernsey | Southern Europe | Serbia |
| Caribbean | Cayman Islands | Eastern Asia | Korea, Dem. People's Rep | Northern Europe | Jersey | Southern Europe | Slovenia |
| Caribbean | Cuba | Eastern Asia | Korea, Republic of | Northern Europe | Isle of Man | Southern Europe | Spain |
| Caribbean | Dominica | Eastern Asia | China, Macao SAR | Polynesia | American Samoa | Southern Europe | Macedonia, Fmr Yug Rp of |
| Caribbean | Dominican Republic | Eastern Asia | Mongolia | Polynesia | Cook Islands | Southern Europe | Yugoslavia SFR |
| Caribbean | Grenada | Eastern Europe | Bulgaria | Polynesia | French Polynesia | Southern Europe | Serbia and Montenegro |
| Caribbean | Guadeloupe | Eastern Europe | Belarus | Polynesia | Niue | Western Africa | Cabo Verde |
| Caribbean | Haiti | Eastern Europe | Czechoslovakia | Polynesia | Pitcairn Islands | Western Africa | Benin |
| Caribbean | Jamaica | Eastern Europe | Czechia | Polynesia | Tokelau | Western Africa | Gambia |
| Caribbean | Martinique | Eastern Europe | Hungary | Polynesia | Tonga | Western Africa | Ghana |
| Caribbean | Montserrat | Eastern Europe | Moldova, Republic of | Polynesia | Tuvalu | Western Africa | Guinea |
| Caribbean | Netherlands Antilles | Eastern Europe | Poland | Polynesia | Wallis and Futuna Is. | Western Africa | Côte d'Ivoire |
| Caribbean | Curaçao | Eastern Europe | Romania | Polynesia | Samoa | Western Africa | Liberia |
| Caribbean | Aruba | Eastern Europe | Russian Federation | South America | Argentina | Western Africa | Mali |
| Caribbean | Sint Maarten | Eastern Europe | Slovakia | South America | Bolivia (Plurinat.State) | Western Africa | Mauritania |
| Caribbean | Bonaire/S.Eustatius/Saba | Eastern Europe | Ukraine | South America | Bouvet Island | Western Africa | Niger |
| Caribbean | Puerto Rico | Melanesia | Solomon Islands | South America | Brazil | Western Africa | Nigeria |
| Caribbean | Saint Barthélemy | Melanesia | Fiji | South America | Chile | Western Africa | Guinea-Bissau |
| Caribbean | Saint Kitts and Nevis | Melanesia | New Caledonia | South America | Colombia | Western Africa | Saint Helena |
| Caribbean | Anguilla | Melanesia | Vanuatu | South America | Ecuador | Western Africa | Senegal |
| Caribbean | Saint Lucia | Melanesia | Papua New Guinea | South America | Falkland Is.(Malvinas) | Western Africa | Sierra Leone |
| Caribbean | Saint-Martin | Micronesia | Kiribati | South America | SouthGeorgia/Sandwich Is | Western Africa | Togo |
| Caribbean | Saint Vincent/Grenadines | Micronesia | Guam | South America | French Guiana | Western Africa | Burkina Faso |
| Caribbean | Trinidad and Tobago | Micronesia | Nauru | South America | Guyana | Western Asia | Azerbaijan |
| Caribbean | Turks and Caicos Is. | Micronesia | Northern Mariana Is. | South America | Paraguay | Western Asia | Bahrain |
| Caribbean | US Virgin Islands | Micronesia | US Minor Outlying Is. | South America | Peru | Western Asia | Armenia |
| Central America | Belize | Micronesia | Micronesia, Fed.States of | South America | Suriname | Western Asia | Cyprus |
| Central America | Costa Rica | Micronesia | Marshall Islands | South America | Uruguay | Western Asia | Georgia |
| Central America | El Salvador | Micronesia | Palau | South America | Venezuela, Boliv Rep of | Western Asia | Palestine, Occupied Tr. |
| Central America | Guatemala | Middle Africa | Angola | South-Eastern Asia | Brunei Darussalam | Western Asia | Iraq |
| Central America | Honduras | Middle Africa | Cameroon | South-Eastern Asia | Myanmar | Western Asia | Israel |
| Central America | Mexico | Middle Africa | Central African Republic | South-Eastern Asia | Cambodia | Western Asia | Jordan |
| Central America | Nicaragua | Middle Africa | Chad | South-Eastern Asia | Indonesia | Western Asia | West Bank |
| Central America | Panama | Middle Africa | Congo | South-Eastern Asia | Lao People's Dem. Rep. | Western Asia | Kuwait |
| Central Asia | Kazakhstan | Middle Africa | Congo, Dem. Rep. of the | South-Eastern Asia | Malaysia | Western Asia | Lebanon |
| Central Asia | Kyrgyzstan | Middle Africa | Equatorial Guinea | South-Eastern Asia | Philippines | Western Asia | Oman |
| Central Asia | Tajikistan | Middle Africa | Gabon | South-Eastern Asia | Timor-Leste | Western Asia | Qatar |
| Central Asia | Turkmenistan | Middle Africa | Sao Tome and Principe | South-Eastern Asia | Singapore | Western Asia | Saudi Arabia |
| Central Asia | Uzbekistan | Northern Africa | Algeria | South-Eastern Asia | Viet Nam | Western Asia | Yemen, Democratic |
| Eastern Africa | British Indian Ocean Ter | Northern Africa | Libya | South-Eastern Asia | Thailand | Western Asia | Syrian Arab Republic |
| Eastern Africa | Burundi | Northern Africa | Morocco | Southern Africa | Botswana | Western Asia | United Arab Emirates |
| Eastern Africa | Comoros | Northern Africa | Sudan | Southern Africa | Lesotho | Western Asia | Turkey |
| Eastern Africa | Mayotte | Northern Africa | Western Sahara | Southern Africa | Namibia | Western Asia | Yemen Arab Republic |
| Eastern Africa | Ethiopia PDR | Northern Africa | Sudan (former) | Southern Africa | South Africa | Western Asia | Yemen |
| Eastern Africa | Ethiopia | Northern Africa | Tunisia | Southern Africa | Swaziland | Western Europe | Austria |
| Eastern Africa | Eritrea | Northern Africa | Egypt | Southern Asia | Afghanistan | Western Europe | Belgium |
| Eastern Africa | French Southern Terr | Northern America | Bermuda | Southern Asia | Bangladesh | Western Europe | Belgium-Luxembourg |
| Eastern Africa | Djibouti | Northern America | Canada | Southern Asia | Bhutan | Western Europe | France |
| Eastern Africa | Kenya | Northern America | Greenland | Southern Asia | Sri Lanka | Western Europe | Germany |
| Eastern Africa | Madagascar | Northern America | St. Pierre and Miquelon | Southern Asia | India | Western Europe | Germany,New Länder |
| Eastern Africa | Malawi | Northern America | United States of America | Southern Asia | Iran (Islamic Rep. of) | Western Europe | Germany, Fed. Rep. of |
| Eastern Africa | Mauritius | Northern Europe | Denmark | Southern Asia | Maldives | Western Europe | Liechtenstein |
| Eastern Africa | Mozambique | Northern Europe | Estonia | Southern Asia | Nepal | Western Europe | Luxembourg |
| Eastern Africa | Réunion | Northern Europe | Faroe Islands | Southern Asia | Pakistan | Western Europe | Monaco |
| Eastern Africa | Rwanda | Northern Europe | Finland | Southern Europe | Albania | Western Europe | Netherlands |
| Eastern Africa | Seychelles | Northern Europe | Ã…land Islands | Southern Europe | Andorra | Western Europe | Switzerland |
| Eastern Africa | Somalia | Northern Europe | Iceland | Southern Europe | Bosnia and Herzegovina |  |  |

**Supplementary Figures**


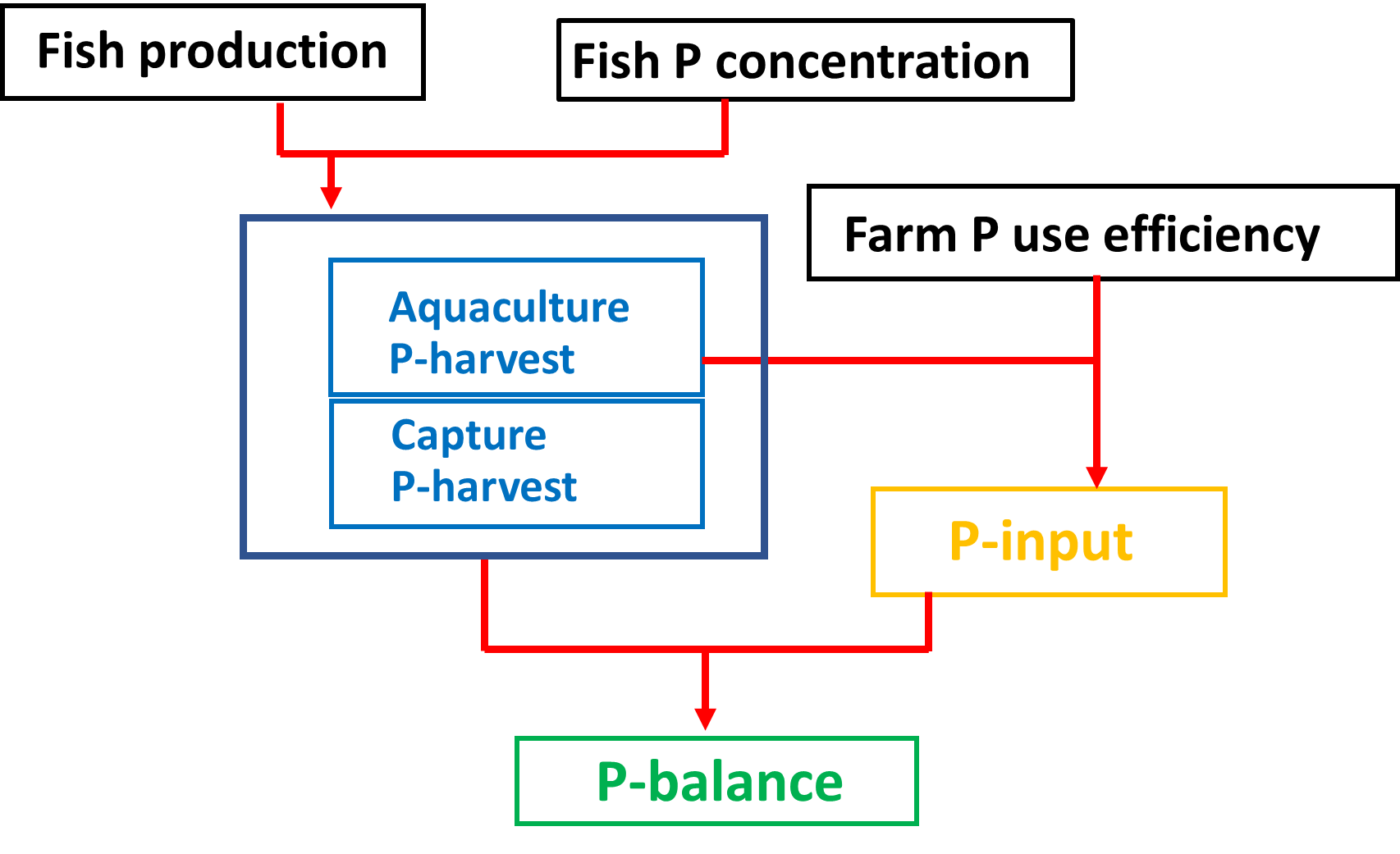


**Supplementary Fig. 15.** **Schematic diagram of fishery P budget calculation.** Black boxes represent data used in the calculation. Derived fluxes are P-harvest, P-input and P-net (i.e., P-harvest – P-input).

**Supplementary Reference**

1 Jana, B. B. Sewage-fed aquaculture: The Calcutta model. *Ecological Engineering* **11**, 73-85, doi:10.1016/s0925-8574(98)00024-x (1998).

2 Kumar, D., Chaturvedi, M. K. M., Sharma, S. K. & Asolekar, S. R. Sewage-fed aquaculture: a sustainable approach for wastewater treatment and reuse. *Environmental Monitoring and Assessment* **187**, doi:10.1007/s10661-015-4883-x (2015).

3 Shepherd, C. J. & Jackson, A. J. Global fishmeal and fish-oil supply: inputs, outputs and markets. *Journal of Fish Biology* **83**, 1046-1066, doi:10.1111/jfb.12224 (2013).

4 Tacon, A. G. J. & Metian, M. Feed matters: satisfying the feed demand of aquaculture. *Reviews in Fisheries Science & Aquaculture* **23**, 1-10, doi:10.1080/23308249.2014.987209 (2015).

5 IPCC. Climate Change 2007: The Physical Science Basis. Contribution of Working Group I to the Fourth Assessment Report of the Intergovernmental Panel on Climate Change [Solomon, S., D. Qin, M. Manning, Z. Chen, M. Marquis, K.B. Averyt, M. Tignor and H.L. Miller (eds.)]. Cambridge University Press, Cambridge, United Kingdom and New York, NY, USA, 996 pp. (2007).

6 Bouwman, A. F. *et al.* Hindcasts and future projections of global inland and coastal nitrogen and phosphorus loads due to Finfish aquaculture. *Reviews in Fisheries Science* **21**, 112-156, doi:10.1080/10641262.2013.790340 (2013).

7 Michielsens, C. G. J., Lorenzen, K., Phillips, M. J. & Gauthier, R. Asian carp farming systems: towards a typology and increased resource use efficiency. *Aquaculture Research* **33**, 403-413, doi:10.1046/j.1365-2109.2002.00686.x (2002).

8 Bouwman, L. *et al.* Exploring global changes in nitrogen and phosphorus cycles in agriculture induced by livestock production over the 1900-2050 period. *Proceedings of the National Academy of Sciences of the United States of America* **110**, 20882-20887, doi:10.1073/pnas.1012878108 (2013).

9 Peñuelas, J. *et al.* Human-induced nitrogen-phosphorus imbalances alter natural and managed ecosystems across the globe. *Nature Communications* **4**, doi:10.1038/ncomms3934 (2013).

10 Chen, M. P. & Graedel, T. E. A half-century of global phosphorus flows, stocks, production, consumption, recycling, and environmental impacts. *Global Environmental Change-Human and Policy Dimensions* **36**, 139-152, doi:10.1016/j.gloenvcha.2015.12.005 (2016).

11 Lun, F. *et al.* Global and regional phosphorus budgets in agricultural systems and their implications for phosphorus-use efficiency. *Earth System Science Data* **10**, 1-18, doi:10.5194/essd-10-1-2018 (2018).
