# Supplementary material for: The shift of phosphorus transfers in global fisheries and aquaculture
Source: Nat Commun. 2020 Jan 17;11:355. doi: 10.1038/s41467-019-14242-7 (PMC6969157; doi:10.1038/s41467-019-14242-7)
Supplement: Supplementary file 3 — Reporting Summary [file 41467_2019_14242_MOESM3_ESM.pdf]

nature research

Corresponding author(s): Yuanyuan Huang

Last updated by author(s): 2019/12/09

Reporting Summary

Nature Research wishes to improve the reproducibility of the work that we publish. This form provides structure for consistency and transparency in reporting. For further information on Nature Research policies, see Authors & Referees and the Editorial Policy Checklist.

Statistics

For all statistical analyses, confirm that the following items are present in the figure legend, table legend, main text, or Methods section.

n/a	Confirmed	
☐	☒	The exact sample size (n) for each experimental group/condition, given as a discrete number and unit of measurement
☒	☐	A statement on whether measurements were taken from distinct samples or whether the same sample was measured repeatedly
☐	☒	The statistical test(s) used AND whether they are one- or two-sided<br>*Only common tests should be described solely by name; describe more complex techniques in the Methods section.*
☒	☐	A description of all covariates tested
☒	☐	A description of any assumptions or corrections, such as tests of normality and adjustment for multiple comparisons
☐	☒	A full description of the statistical parameters including central tendency (e.g. means) or other basic estimates (e.g. regression coefficient) AND variation (e.g. standard deviation) or associated estimates of uncertainty (e.g. confidence intervals)
☐	☒	For null hypothesis testing, the test statistic (e.g. F, t, r) with confidence intervals, effect sizes, degrees of freedom and P value noted<br>*Give P values as exact values whenever suitable.*
☒	☐	For Bayesian analysis, information on the choice of priors and Markov chain Monte Carlo settings
☐	☒	For hierarchical and complex designs, identification of the appropriate level for tests and full reporting of outcomes
☒	☐	Estimates of effect sizes (e.g. Cohen's d, Pearson's r), indicating how they were calculated

Our web collection on statistics for biologists contains articles on many of the points above.

Software and code

Policy information about availability of computer code

Data collection	WebPlotDigitizer-3.8-Desktop
Data analysis	Python 2.7.15

For manuscripts utilizing custom algorithms or software that are central to the research but not yet described in published literature, software must be made available to editors/reviewers. We strongly encourage code deposition in a community repository (e.g. GitHub). See the Nature Research guidelines for submitting code & software for further information.

Data

Policy information about availability of data

All manuscripts must include a data availability statement. This statement should provide the following information, where applicable:

- Accession codes, unique identifiers, or web links for publicly available datasets
- A list of figures that have associated raw data
- A description of any restrictions on data availability

Databases of fish P concentration, culture system level P use efficiency and P retention efficiency are provided as Supplementary Data. Additional data that support the findings of this study are available from the corresponding author upon request.

Field-specific reporting

Please select the one below that is the best fit for your research. If you are not sure, read the appropriate sections before making your selection.

☐ Life sciences ☐ Behavioural & social sciences ☒ Ecological, evolutionary & environmental sciences

Ecological, evolutionary & environmental sciences study design

All studies must disclose on these points even when the disclosure is negative.

Study description	This study collect extensive dataset from literature and existing dataset to quantify phosphorus budget
Research sample	The dataset is collected from literature and existing databases. References to the literature are provided in Supplementary Data. The existing databases include FishStatJ version 3.04.6 and from Sea Around Us (http://www.seaaroundus.org/)
Sampling strategy	The sampling size is based on the entries that is available from what we can collect from literature
Data collection	Yuanyuan Huang collected literature through ISI web of science and Google scholar. Yuanyuan Huang manually extracted data from literature if it is in a table format and through WebPlotDigitizer-3.8-Desktop for figure fomats
Timing and spatial scale	The collection started from 7th August 2017 to 10th September 2018. The the spatial span covers the whole globe
Data exclusions	NO data was excluded
Reproducibility	We carefully quantified the uncertainty of our approach and made sure the findings are reliable. All data processing are recorded in python script to guarantee reproducibility
Randomization	We collected large samples that are available in literature to do a budget quantification. And this is not a controlled experimental study
Blinding	We collected large samples that are available in literature to do a budget quantification. And this is not a controlled experimental study

Did the study involve field work? ☐ Yes ☒ No

Reporting for specific materials, systems and methods

We require information from authors about some types of materials, experimental systems and methods used in many studies. Here, indicate whether each material, system or method listed is relevant to your study. If you are not sure if a list item applies to your research, read the appropriate section before selecting a response.

Materials & experimental systems

n/a	Involved in the study	
☒	☐	Antibodies
☒	☐	Eukaryotic cell lines
☒	☐	Palaeontology
☒	☐	Animals and other organisms
☒	☐	Human research participants
☒	☐	Clinical data

Methods

n/a	Involved in the study	
☒	☐	ChIP-seq
☒	☐	Flow cytometry
☒	☐	MRI-based neuroimaging
