# Supplementary material for: The shift of phosphorus transfers in global fisheries and aquaculture
Source: Nat Commun. 2020 Jan 17;11:355. doi: 10.1038/s41467-019-14242-7 (PMC6969157; doi:10.1038/s41467-019-14242-7)
Supplement: Supplementary file 4 — Description of Additional Supplementary Files [file 41467_2019_14242_MOESM4_ESM.docx]

**Description of Additional Supplementary Files**

File Name: Supplementary Dataset 1

Description: List of whole body P concentration of fish.

File Name: Supplementary Dataset 2

Description: List of culture system level fishery phosphorus use efficiency.

File Name: Supplementary Dataset 3

Description: List of fishery P retention efficiency.

File Name: Source data

Description: Source data to Figs 2 and 3.
